# Carbenoxolone mitigates extensive fibrosis formation in PLP-induced EAE model and multiple sclerosis serum-exposed pericyte culture

**DOI:** 10.3389/fncel.2024.1403974

**Published:** 2024-04-30

**Authors:** Ege Anil Ucar, Esra Ozkan, Narges Shomalizadeh, Emine Sekerdağ-Kilic, Fatmanur Akpunar, Selin Sapanci, Judy Kesibi, Ceyda Ozler, Alara Su Bilgez, Yasemin Gursoy-Ozdemir

**Affiliations:** ^1^Research Center for Translational Medicine (KUTTAM), Koҫ University, Istanbul, Türkiye; ^2^School of Medicine, Koç University, Istanbul, Türkiye; ^3^Department of Neurology, Koç University, Istanbul, Türkiye

**Keywords:** multiple sclerosis, fibrosis, PLP EAE, pericytes, blood–brain barrier, carbenoxolone

## Abstract

**Introduction:**

Multiple sclerosis (MS) is one of the most common causes of disability in young adults. Nearly, 85% of MS cases start with attacks and remissions, classified as relapsing-remitting multiple sclerosis (RRMS). With repeating attacks, MS causes brain-spinal cord atrophy and enhanced disability as disease progresses. PLP-induced EAE is one of the most established models for pathophysiology and treatment of RRMS. Recent studies demonstrated the possible role of pericytes in perivascular and intra-lesional fibrosis in PLP-induced EAE, whose importance remains elusive. Hence, we have investigated the possible role of pericytes in fibrosis formation and amelioration with a hemichannel blocker, Carbenoxolone (CBX).

**Methods:**

PLP-induced experimental autoimmune encephalitis (EAE) model is used and the effect of CBX is investigated. Clinical scores were recorded and followed. Perivascular Collagen 1 and 3 accumulations were demonstrated as markers of fibrosis in the spinal cord. To delineate the role of pericytes, human brain vascular pericytes (HBVP) were incubated with the sera of MS patients to induce in-vitro MS model and the fibrosis formation was investigated.

**Results:**

In the PLP induced in-vivo model, both intracerebroventricular and intraperitoneal CBX have significantly mitigated the disease progression followed by clinical scores, demyelination, and fibrosis. Moreover, CBX significantly mitigated MS-serum-induced fibrosis in the HBVP cell culture.

**Discussion:**

The study demonstrated two important findings. First, CBX decreases fibrosis formation in both in-vivo and in-vitro MS models. Secondly, it improves neurological scores and decreases demyelination in the EAE model. Therefore, CBX can be potential novel therapeutic option in treating Multiple Sclerosis.

## 1 Introduction

Multiple Sclerosis (MS) is an autoimmune, chronic inflammatory, demyelinating neurological disease of the central nervous system (McGinley et al., 2021) that is the most common non-traumatic disabling disease that affects young adults. The prevalence and incidence of MS are increasing in developed and developing countries (Browne et al., 2014). The majority of the patients exhibit a relapsing–remitting (85%) type of the disease (Ruiz et al., 2019). Aside from this form, there are different clinical presentations, including primary-progressive MS and late-onset MS (Buscarinu et al., 2022). As the disease progresses, cognitive dysfunction and progressive neurological deterioration causes significant disability. Given the cost of new disease-modifying therapies and the duration of the disease, MS causes a considerable burden on society and the individual. Additionally, neurodegeneration and brain volume loss are found to be one of the major contributors to disability progression, independent of disease activity. The mechanism of this neurodegeneration and brain atrophy are understudied and gained insight in recent years (Hamzaoui et al., 2023). MOG and PLP induced experimental autoimmune encephalomyelitis models are commonly used models for mimicking MS. PLP-induced EAE model goes with attacks and remissions, therefore mimicking relapsing–remitting MS (RRMS). On the other hand, MOG-induced EAE model causes a progressive neurological deterioration, hence mimic primary-progressive MS better (Şekerdağ-Kılıç et al., 2023).

Both autoinflammation and gliosis contribute to the development of MS lesions and disease progression, which are well-described. Recent studies suggest that MS lesions primarily consist of a fibrotic core and inflammatory cells in the periphery (Fernández-Klett and Priller, 2014; Dias and Göritz, 2018). Moreover, vessels neighboring MS lesions show perivascular thickening (Mohan et al., 2010). Thus, the importance of perivascular fibrosis in MS pathophysiology needs to be answered.

Pericytes, a neurovascular unit member located around and wrapping the endothelial wall maintaining the blood–brain barrier (BBB), are shown to contribute to scar formation in brain injury models (Arimura et al., 2012). Their count and coverage status are associated with the permeability of the BBB; a lower coverage correlates with a higher permeability (Dore-Duffy, 2003; Girolamo et al., 2019; Heymans et al., 2020). Moreover, pericytes can regulate the capillary structure and diameter (Armulik et al., 2010; Bell et al., 2010; Winkler et al., 2012) and even guide vessel sprouting (Gerhardt and Betsholtz, 2003; Daneman et al., 2010). Pericytes are the first responders to systemic neuroinflammation, and they seek to increase their coverage to maintain BBB in MS (Rafalski et al., 2018; Kaushik et al., 2021). These cells can differentiate into myofibroblasts and mediate fibrosis (Rafalski et al., 2018; Dias et al., 2021; Özkan et al., 2021). The fibrotic role of pericytes was elaborately demonstrated in liver, kidney, and lung fibrosis models and also in dermal scarring (Greenhalgh et al., 2013). However, only a limited number of studies examined the role of pericytes in perivascular fibrosis seen in MS (Brown and Thore, 2011; Dorrier et al., 2021). Recently, our group reported that the pericytes contribute to fibrosis and disease progression in the myelin oligodendrocyte glycoprotein (MOG) and proteolipid protein induced (PLP) experimental autoimmune encephalitis (EAE) models (Şekerdağ-Kılıç et al., 2023). Moreover, our previous studies pointed to the possible role of pericytes especially in perivascular fibrosis (Özkan et al., 2021; Şekerdağ-Kılıç et al., 2023). At the site of the BBB breakdown, perivascular PDGFRβ positive cells (pericytes) accumulate and thicken the vessel wall via extracellular matrix deposition, hence cause fibrosis, especially in PLP-induced EAE model. Thus, in this current study, we want to delineate if prevention of extensive perivascular fibrosis might be protective in the MS animal model (PLP-induced EAE).

Carbenoxolone (CBX) is a disodium salt of the 3-O-hydrogen succinate of glycyrrhetic acid (Uyama et al., 2003). CBX exerts its effects by inhibiting the hemichannels, particularly pannexins. It was shown to be protective in liver and lung fibrosis (Crespo Yanguas et al., 2018; He et al., 2022). It also exerts its anti-fibrotic actions in the pancreatic and hepatic stellate cell cultures, which are very similar to brain pericytes hence they are named as hepatic and pancreatic pericytes (Uyama et al., 2003; Masamune et al., 2013). Previous studies have shown that in the MOG EAE model, CBX treatment delays the onset and decreases the severity of neurological symptoms (Shijie et al., 2009; Endong et al., 2011; Chen et al., 2020). Due to its anti-fibrotic effects as well as effects on satellite cells (pericytes of liver and pancreas) and possible role of fibrosis in EAE models pathophysiology, we want to detect antifibrotic effect of CBX on PLP induced EAE model and define possible therapeutic target. Hence the PLP induced EAE model is conducted in mice. Since CBX has low penetration through the BBB (Leshchenko et al., 2006), local delivery of CBX was done through intracerebroventricular (ICV) cannulas in addition to systemic (i.p.) administration. Moreover, cultured human brain vascular pericytes were exposed to the sera of the patients diagnosed with relapsing–remitting MS that were collected at the attack phase to model the inflammatory milieu of MS *in-vitro*.

## 2 Materials and methods

### 2.1 Animals and human material

8–12 weeks old 22 female BalbC mice were used. All animals were housed in a controlled environment with *ad libitum* access to food and water, 12 h of light/dark cycle, and 20 ± 3°C. All experiments and procedures were applied according to the protocols and criterions approved by the Institutional Animal Care and Use Committee (IACUC) at Koç University (2016–02, 2022–11 approval numbers) and according to Directive 2010/63/EU of the European Parliament and the Council on the Protection of Animals Used for Scientific Purposes. All experiments were reported in compliance with the animal research: reporting *in-vivo* experiments (ARRIVE) guidelines. All measures were taken to reduce the suffering of the animals.

The Institutional Review Board (IRB) of Koç University reviewed and approved the use of human serum in *in-vitro* experiments (approval number: 2016.123.IRB2.077). Informed consent was obtained from all subjects, and the study has been performed in accordance with the Declaration of Helsinki. The sera were collected from three healthy controls and at the attack period from three patients diagnosed with relapsing–remitting type MS by a neurologist and validated by cerebrospinal fluid (CSF)-specific oligoclonal bands used for this study.

### 2.2 PLP EAE animal model

**Figure 1:**
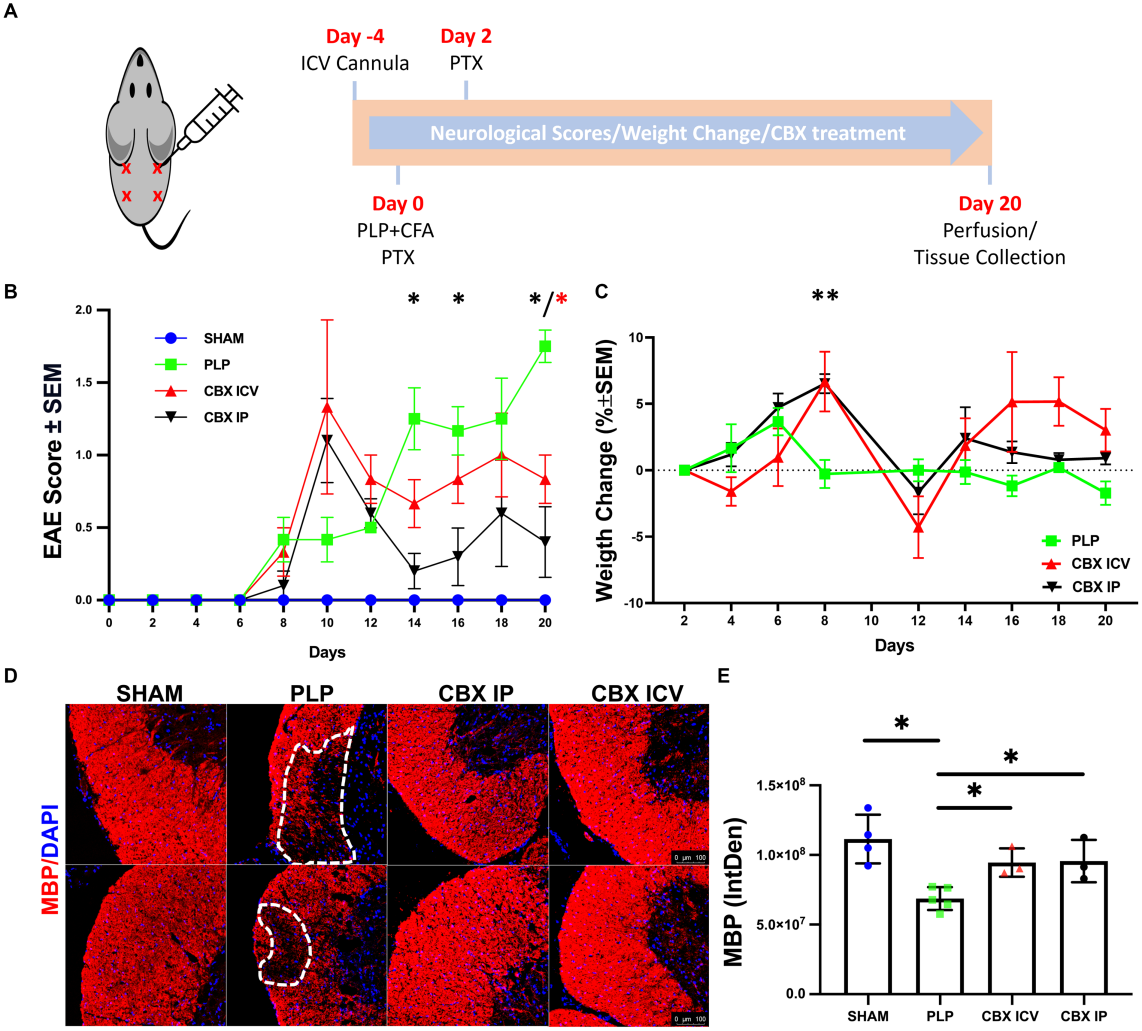
Validation of PLP-EAE model. **(A)** The injection sides and the timeline of the experiment are shown. **(B)** The neurological scores deteriorated quickly at day 8 and entered remission from day 12 to the end of the experiment for CBX-treated groups but increased gradually and were maximum on the last day of the investigation for the PLP-EAE group. **(C)** Weight gain followed a similar trend with the neurological scores, and the worst day (the maximum weight loss) was observed after the day with top neurological scores. **(D)** Myelin Basic Protein (MBP)-stained spinal cord sections were shown. The lesion areas were marked with dashed lines in the PLP group. The scale bar is 100 μm. **(E)** The fluorescent intensity of MBP was significantly decreased in the PLP group compared to the other groups and both IP and ICV CBX treatment decreased demyelination (SHAM: 40 region, *n* = 4; PLP: 85 region, *n* = 5, CBX ICV: 24 region, *n* = 3; CBX IP: 21 region, *n* = 3). Data were presented as Mean ± SEM for **(B,C)**, and Mean ± SD for E. The significance of comparisons for PLP-EAE versus IP CBX was shown with black asterisks and ICV CBX versus PLP-EAE with red asterisks. *p < 0.05, ** < 0.01. IntDen, integrated density.

The relapsing–remitting MS model was created by immunizing female BalbC mice with PLP antigen (Figure 1A). For each animal, 100 μL of PLP139-151 dissolved in PBS at 1 mg/mL concentration (PLP-3812-PI from Peptides International, Louisville, KY) was emulsified in 100 μL of complete Freund’s adjuvant (CFA) including 5.33 mg/mL of *M. tuberculosis* H37. Under isoflurane anesthesia, a total of 200 μL was divided into four locations and administered subcutaneously. In addition, 250 ng of 5µg/mL Pertussis toxin (PTX) (cat. no. P7208-50UG, Sigma-Aldrich) has been injected intraperitoneally to each mouse 1 h and 2 days after the vaccination.

The experimental groups were Sham (*n* = 6), PLP EAE (*n* = 6), CBX IP (*n* = 5), and CBX ICV (*n* = 5). The sham group received the CFA adjuvant only without PLP. The CBX IP group received CBX at a dose of 20 mg/kg from 2.5 mg/mL intraperitoneally, and the CBX ICV group 25 μg/kg from 0.25 mg/mL intracerebroventricularly from the cannula every other day starting from day 0. All experimental animals’ weight changes and EAE scores were monitored every 2 days for 20 days. Evaluation of EAE clinical results comprised body weight measurement and clinical EAE rating of the tail and limbs (0–5 scale): 0 = Healthy, 1 = Flaccid tail, 2 = Ataxia or paralysis of hind limbs, 3 = Paralysis of hind limbs and/or ataxia or paralysis of forelimbs, 4 = Ataxia or paralysis of all limbs, 5 = Dead.

### 2.3 Surgery

An intracerebroventricular cannula was placed on the CBX-ICV group (one animal died during surgery). After anesthesia with ketamine/xylazine (100/10 mg/kg, i.p), animals were placed in the stereotactic frame, and the skull was cleaned with 70% ethanol. A midline incision was made, and a burr hole was opened gently with a drill at the coordinate 0.1 mm posterior and 0.9 mm left to the bregma. An i.c.v. cannula was placed through the burr hole. It was stabilized with nontoxic jealous glue from the sides of the skull and surrounded with dental cement for further stabilization.

### 2.4 Perfusion

On day 20 of immunization, the mice were sacrificed with an overdose of Ketamine/Xylazine, and cardiac blood samples were taken from each animal. Then they were cardiac perfused with ice-cold saline solution and 4% paraformaldehyde (PFA). Brains and spinal cords were immediately removed and incubated in 4% PFA at 4°C for at least 24 h and in 10, 20, and 30% sucrose (Caisson #SO11) solutions until each tissue settled to the bottom of the Falcon tube. Tissues were frozen at the optimal cutting temperature (OCT, Leica, 14,420,108,926) and stored until use at −80°C. On poly-l-lysine-coated, positively charged glass slides, 10-μm-thick frozen brain slices were collected from these brain and spinal cord samples.

### 2.5 Immunofluorescence staining

The IF protocol was explained elsewhere in detail (Özkan et al., 2021). The primary antibodies used in this study were Mouse anti-myelin-basic protein (anti-MBP) (1:200, Ab62631), Rabbit anti-collagen 1 (1:500, Ab34710), Rabbit anti-collagen 3 (1:500, Ab7778), Mouse anti-smooth muscle actin-FITC conjugated (1:100, F3777), and Rabbit anti-fibronectin (1:500, F3648). Sections were mounted with 4′ 6-diamidino-2-phenylindole (DAPI, Abcam, ab104139). We divided the spinal cord into five parts axially and at least 2 sections per part were taken for each animal. The image analysis was conducted for *n* = 14–90 per group. All photos obtained within each experiment were subjected to the same acquisition parameters with Fluorescent (DMI8; Leica) or confocal (DMI8/SP8; Leica) microscopes. The images were exported with LASX software and analyzed with ImageJ software as previously described (Özkan et al., 2023).

### 2.6 Cell culture

Human brain vascular pericytes (HBVP, ScienceCell, 1,200) were cultured on poly-L-lysine (PLL, Sigma-Aldrich, P2658) coated 96-well plate. HBVPs were incubated at 37°C and 5% CO_2_ until the confluency reached 2 × 10^4^ cells/well in 200 μL. The pericyte medium (Science cell, USA) supplemented with Pericytes growth supplement (Science cell, USA) was used because the HBVP are fragile and do not proliferate in regular culture mediums.

*Plates wells were grouped as follows:* control-blank (only medium), treatment (medium + treatment), and serum-treatment (medium + treatment + serum). Twenty-four hours after cultivation in the 96 wells-plate, 10 μL of the medium from the serum-treatment wells were discarded. The treatments and MS patients’ serum were consecutively added to the related wells. Each treatment substance was repeated for three different serum specimens; all samples were duplicated.

The substances added to the treatment and serum-treatment wells were Methylprednisolone (Catalog No: M0639, Sigma-Aldrich) (10 mM in DMSO) and carbenoxolone (Catalog No: C4790, Sigma-Aldrich) (10 mM in PBS: DMSO). The added volumes from the treatments were 2.8 μL and 1 μL, respectively. Then, 10 μL of the MS serums were added to the serum-treatment wells.

The well plate was incubated for 24 h in the same incubation conditions. Next, the supernatants were removed and immediately frozen for further ELISA and hydroxyproline assay studies. Then, the wells were washed with DPBS, and 100 μL of 4% paraformaldehyde was added to each well; the plate was then incubated at room temperature. After 20 min, the supernatant was removed, the wells were washed with DPBS, and continued to the IF staining protocol. For PCR studies, the cells at predefined wells were incubated with 0.05% trypsin for 5 min at 37^0^C. The detached cells suspended in trypsin solution were centrifuged at 1500 rpm (363 g) for 5 min, and the pellet was collected.

### 2.7 RT-qPCR

Quick-RNA™ Microprep kit (Zymo Research, R1054 & R1055) and cDNA Synthesis kit (Bip-rad, BR1708891) were used for RNA extraction and cDNA synthesis, respectively. The experiment was conducted on a LightCycler 480 II (Roche) using LightCycler® 480 SYBR Green I Master mix (Roche, cat. no. 14571520). The primers for the Col3a1 gene were (5′ 3′ forward, TTG AAG GAG GAT GTT CCC ATC T) and (5′ 3′ reverse, ACA GAC ACA TAT TTG GCA TGG TT). Annealing, denaturation, and extension temperature were 60°C, 94°C, and 72°C, respectively.

### 2.8 ELISA

Elisa plates were coated with media collected from the HBVP cell culture or serum of animals from the *in-vivo* MS model in PBS and incubated overnight at 4°C. Plates were blocked by 5% BSA in PBS for 2 h at room temperature. Then, 100 μL of Rabbit anti-fibronectin antibody (1:10000) in 2% BSA-PBS was added to the plate and incubated at 37°C for 90 min. After that, 100 μL anti-RB HRP (1:10000) antibodies in 2% BSA-PBS were added to the plate and incubated for 90 min at room temperature. Following washing, 100 μL of 3.3′, 5.5′-tetramethylbenzidine was added to wells and incubated for 10 min. 100 μL of 1 M HCL was used to stop the reaction. For validation of the animal model, the plates were coated with PLP antibodies and then incubated with the serum collected from the animals. Optical density values were measured at 450 nm.

### 2.9 Hydroxyproline collagen assay

100 μL of media from the HBVP experiments were added to a 2 mL cryovial. 100 μL of 4 M NaOH was added to the cryovials. Then, vials were autoclaved at 120°C 15 psi for 30 min. After allowing samples to return to RT, 100 μL of 4 N HCL was added to naturalize the PH. 625 μL of Chloramine T solution was added, and samples were incubated at RT for 20 min. 625 μL of Ehrlich’s solution was added and incubated in a water bath of 65°C for 20 min (Cissell et al., 2017). Finally, 200 μL of samples and standards were added to a 96-well plate, and the absorbance was measured at 550 nm.

### 2.10 Statistical analysis

All statistical analyses were done with GraphPad Prism software 8.4.3 (GraphPad Software Inc., La Jolla, CA, United States). Weight changes and EAE score were presented as Mea*n* ± SEM. All other data were presented as Mean ± SD. Nonparametric comparisons betweens groups were made with the Kruskal-Wallis test and in between groups with the Mann–Whitney U test for *in vivo* model’s immunofluorescence intensity and area analysis and all *in-vitro* model’s analysis. For *in vivo* clinical scores and weight changes, mixed-model two-way ANOVA analysis was used. A *p*-value smaller than 0.05 was accepted as significant.

### 2.11 Study approval

All experiments and procedures were applied according to the protocols and criterions approved by the Institutional Animal Care and Use Committee (IACUC) at Koc University (2016–02, 2022–11 approval numbers) and according to Directive 2010/63/EU of the European Parliament and the Council on the Protection of Animals Used for Scientific Purposes. The Institutional Review Board (IRB) of Koç University reviewed and approved the use of human serum in *in-vitro* experiments (approval number: 2016.123.IRB2.077). Informed consent was obtained from all subjects, and the study has been performed in accordance with the declaration of Helsinki.

## 3 Results

### 3.1 Validation of the animal model and effect of CBX on clinical outcome

The PLP-induced EAE model is validated across groups with weight and clinical score follow-up. After the termination of experiments, serum anti-PLP antibody levels were measured with ELISA, and the demyelinated MS lesions were shown with myelin-basic protein (MBP) immunofluorescence staining.

The neurological deficits started around day 8 for all EAE-induced animals, Figure 1B. For the CBX-treated groups, neurological scores deteriorated quickly at day 10 and entered remission from day 12 to the end of the experiment. However, for the untreated PLP-EAE group, the neurological deficits increased gradually and reached a maximum level on the last day (Day 20) of the investigation. In parallel with this, statistical analyses revealed that ICV and IP CBX-treated groups had significantly lower scores than the PLP-only group at day 20 (*p* = 0.036, *p* = 0.011) respectively. Moreover, IP CBX group exhibited better EAE scores on day 14 and 16 (*p* = 0.012, *p* = 0.040) respectively. The Sham group did not show any neurological deficits during follow-up.

The percentage of weight gain regarding the previous measurement was calculated for all animals, Figure 1C. Weight gain followed a similar trend with the neurological scores, and the worst day (the maximum weight loss) was observed after the day with top neurological scores. The CBX IP group continued to gain weight until day eight similar to the PLP-EAE group, which stopped on day 6. So, on day 8, weight change was significantly higher in the IP CBX-treated group (*p* = 0.0015). Moreover, toward the end of the follow-up both ICV CBX and IP CBX groups gained more weight than PLP group, however this does not reach significance.

After completing the experiment, anti-PLP antibody levels were measured in animal serum samples of the sham and PLP groups (*n* = 5–6) using the ELISA method to prove that the EAE model is successful. The optical density of the sham group was 0.08 ± 0.02, and for the PLP group, it was 2.62 ± 0.79. As expected, the anti-PLP antibody levels were significantly higher in the PLP group (*p* = 0.004).

To further delineate the effect of CBX on demyelination, spinal cord sections were stained for MBP. The lesion areas were clearly visualized in the PLP EAE group but not in the CBX-treated groups, Figure 1D. Moreover, the fluorescent intensity of MBP-stained areas was significantly different between groups (*p* = 0.013). The PLP group had lower fluorescence intensity, defined as integrated density, values compared to the sham, CBX ICV, and CBX IP groups (*p* = 0.001, *p* = 0.036, and *p* = 0.036) respectively. Notably, both CBX ICV and CBX IP groups showed similar and significant improvement in demyelinated areas, Figure 1E.

### 3.2 Collagen 3 expression is increased in the PLP EAE model in spinal cord sections and ICV CBX application mitigated the extensive fibrosis

**Figure 2:**
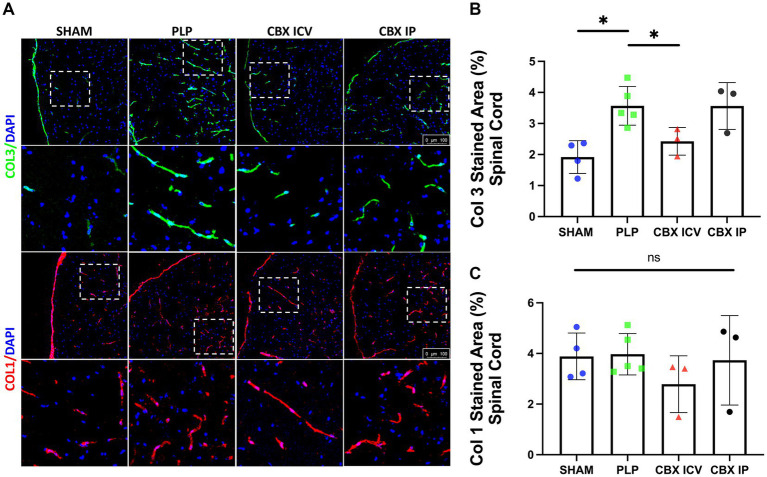
Fibrosis in EAE model. **(A)** Collagen 3 (green, first row) and Collagen 1 (red, 3rd row) stained spinal cord sections from each group are shown. The closer images of the areas marked with dashed rectangles were shown below the rows. Collagen 3 expression clearly increased in the PLP-only group. **(B)** The image analysis results demonstrated that Collagen 3 stained area is significantly increased in the PLP group and successfully reduced in the CBX-ICV group (SHAM: 38 region, *n* = 4, PLP: 76 region, *n* = 5, CBX ICV: 27 region, *n* = 3, CBX IP: 24 region, *n* = 3). **(C)** The Collagen 1 stained area was similar between groups (SHAM: 28 region, *n* = 4, PLP: 30 region, *n* = 5, CBX ICV: 24 region, *n* = 3; CBX IP: 21 region, *n* = 3). The scale bar is 100 μm. Data were presented as Mean ± SD. NS: non-significant, *p < 0.05.

The Collagen 3 and Collagen 1 expressions in spinal cord sections were studied with IF staining, Figure 2. With induction of the EAE model, significant perivascular collagen accumulation was detected. The Collagen 3 expression differed significantly between groups (*p* = 0.002). The PLP EAE group showed significant perivascular overexpression of Collagen 3 than the sham and CBX ICV groups (*p* = 0.016, *p* = 0.036, respectively), Figures 2A,B. Notably, IP treatment of CBX did not exert any effect on PLP EAE model-induced Collagen 3 accumulation (*p* > 0.999). On the other hand, perivascular collagen 1 expression was not significantly different between PLP EAE and treatment groups (*p* = 0.609), Figures 2A–C.

### 3.3 Exposure to MS serum leads to the overproduction of fibrotic elements in pericyte culture, which can be mitigated by CBX

In addition to collagen 3, we wanted to evaluate the role of pericytes in perivascular fibrosis in MS pathophysiology, human brain vascular pericytes (HVBP) were exposed to MS sera collected from three patients with relapsing–remitting MS at the attack period, and three healthy control sera. CBX and methylprednisolone (MP) were tested for their anti-fibrotic effect. After the incubation, the supernatant was quickly removed and used to quantify total Collagen and Fibronectin levels. In addition, the cells were collected, and the Col3a1 mRNA expression was studied.

**Figure 3:**
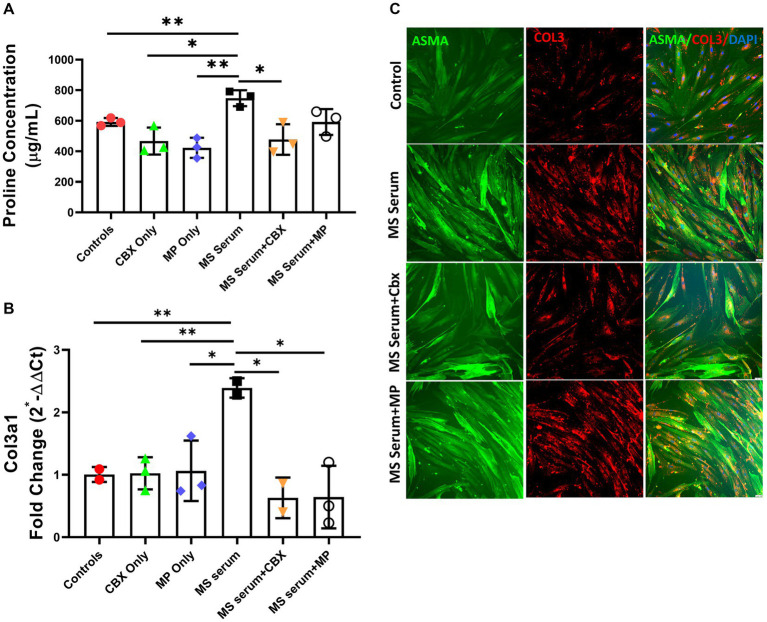
Collagen 3 expressions in pericyte culture treated with MS serum. **(A)** Total proline concentration was measured with collagen assay. MS serum significantly increased proline concentration. And treatment with CBX but not MP decreased MS serum-induced overproduction. **(B)** Col3a1 mRNA expression was studied with qPCR. Incubation with MS serum significantly increased the Collagen 3 gene expression. **(C)** The first column shows Alpha Smooth Muscle Actin (ASMA)-stained pericytes in green, the second column shows Collagen 3 in red, and the third column represents the overlapped image with the DAPI-stained blue cell nucleus. MS serum exposure increased the collagen 3 staining more than controls (second row), and treatment with CBX but not MP decreased the MS Serum effect on Collagen 3 production. The scale bar is 100 μm. Data are presented as Mea*n* ± SD. * ≤ 0.05, ** < 0.01.

The total proline concentration, used to demonstrate total collagen production, was significantly higher in MS patients’ sera incubated wells (*p* = 0.002) (MS Serum: 747.6 ± 52.1, MS Serum+CBX: 477.0 ± 100.0, MS Serum+MP: 592.0 ± 84.5 μg/mL, Control: 592.0 ± 25.5, CBX Only: 466.8 ± 88.5, MP Only: 422.8 ± 66.0, *n* = 3 per group), Figures 3A,B. Exposure to the MS sera significantly increased the extracellular Collagen concentration produced by pericytes, compared to the control wells (*p* = 0.009). Application of CBX decreased serum-induced fibrosis (*p* = 0.014). On the other hand, application of MP also lowered the Collagen levels in the supernatant, but the difference did not reach significance (*p* = 0.053).

Since we detected increased perivascular collagen 3 production *in vivo*, we wanted to see collagen 3 gene expression changes in the HBVP culture. Incubation with MS sera significantly increased Collagen 3 gene expression (*p* = 0.008). CBX and MP applications both decreased the overexpression induced by MS sera incubation (*p* = 0.020, *p* = 0.019). Neither CBX nor MP did not change the collagen 3 basal expression level (MS Serum: 2.39 ± 0.16, MS Serum+CBX: 0.63 ± 0.22, MS Serum+MP: 0.64 ± 0.50, Control: 1.01 ± 0.12, CBX Only: 1.02 ± 0.26, MP Only: 1.06 ± 0.48, fold change, *n* = 2–3 per group), Figures 3A–C.

**Figure 4:**
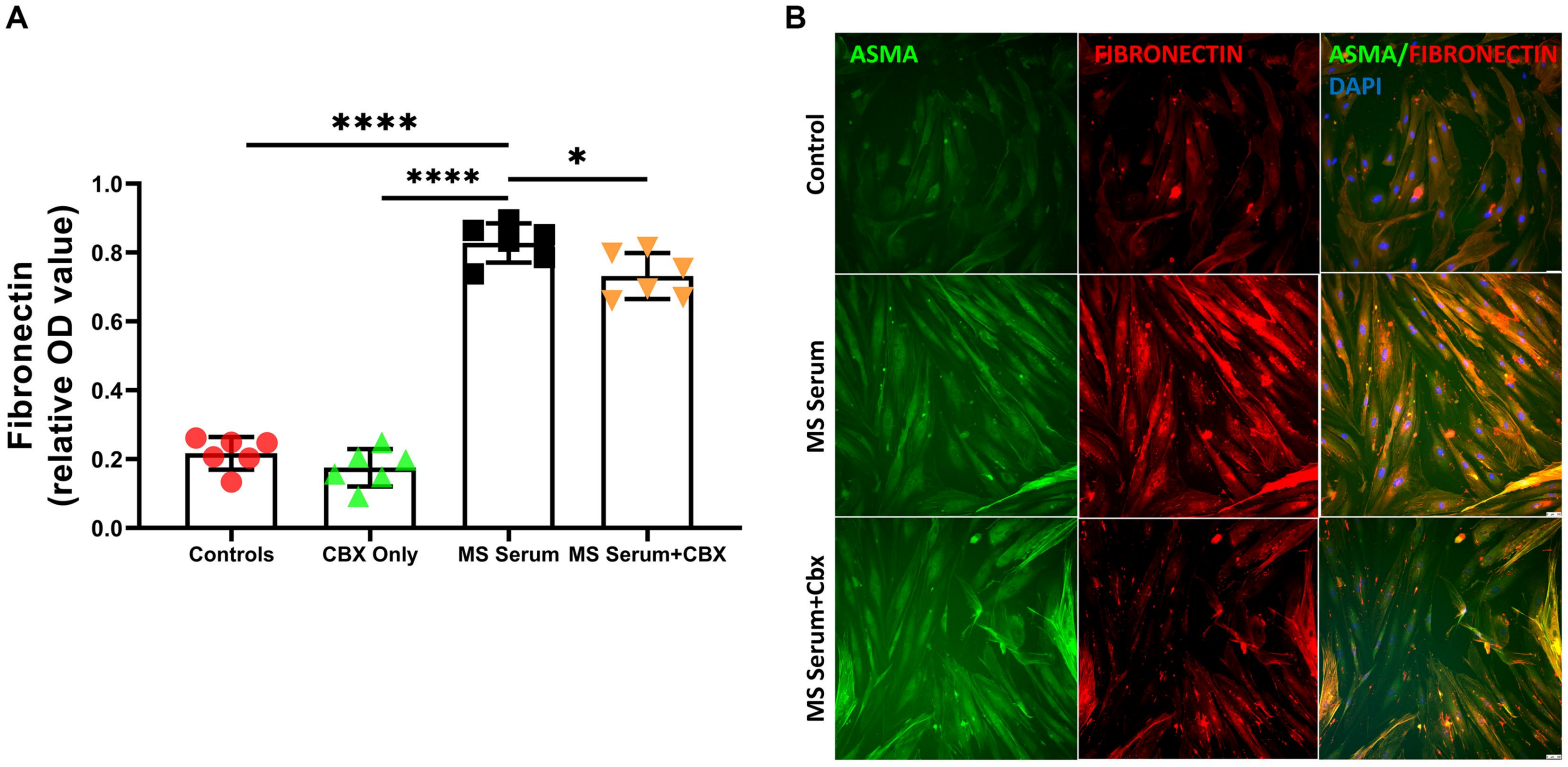
Fibronectin expressions in pericyte culture treated with MS serum. **(A)** Fibronectin levels were measured with ELISA. MS Serum significantly increased extracellular Fibronectin, and CBX treatment decreased this over-synthesis. **(B)** The first column shows Alpha Smooth Muscle Actin (ASMA)-stained pericytes in green, the second column shows Fibronectin in red, and the third column represents the overlapped image with the DAPI-stained blue cell nucleus. MS serum exposure increased the Fibronectin staining more than controls (second row), and treatment with CBX decreased the MS Serum effect on Fibronectin production. The scale bar is 100 μm. Data are presented as Mean ± SD. * ≤ 0.05, *** < 0.001, **** < 0.0001. OD, optical density.

Since perivascular pericytes have ability to produce increased amounts of fibronectin as a reaction to insults (Mandarino et al., 1993; Makihara et al., 2015) and fibronectin have a possible role in regulating endothelial permeability to inflammatory cells (Sava et al., 2015), we evaluated the effect of MS sera and CBX incubation on fibronectin levels. Fibronectin levels were measured with ELISA. The fibronectin levels after incubation with MS sera were significantly increased (*p* < 0.0001). Moreover, this increase was ameliorated with CBX application (*p* = 0.05). (MS Serum: 0.86 ± 0.03, MS Serum+CBX: 0.79 ± 0.03, Control: 0.25 ± 0.01, CBX Only: 0.20 ± 0.01, OD, *n* = 3 per group), Figures 4A,B.

## 4 Discussion

The role of perivascular cells and blood–brain barrier (BBB) in MS pathophysiology is a newly recognized area and a limited number of studies have been done until today. We have previously shown pronounced perivascular fibrosis as well as increased number of pericytes that are proliferating around MS lesions and expressing myofibroblastic transformation marker alpha smooth muscle actin (ASMA) *in vivo* PLP EAE model. This reaction might be related to myofibroblastic change of pericytes (Şekerdağ-Kılıç et al., 2023). We can speculate that this transformation may be leading to perivascular fibrosis formation. However, there was a need for demonstration of pericyte involvement in MS-induced extracellular matrix production and its mitigation. Hence, we conducted the PLP EAE model and used *in vitro* pericyte culture model with MS patient sera incubation and compared the reactions of pericytes in both conditions. We have chosen PLP-induced EAE model since it mimicks RRMS, which accounts for 85% of MS patients’ first attack. Our results demonstrated that pericytes behaved in the same manner, in both *in vivo* (animal model) and *in vitro* (human model). Total collagen and collagen 3 protein levels were increased in both conditions. As a next step, we want to demonstrate involvement of pericytes in fibrosis-extracellular matrix production and to further delineate the mechanism involved. In order to do that, we took the advantage of previously tested and effective molecules in MS models and MS clinical attacks. Methylprednisolone is one of the most effective and well-established treatments of attacks. And CBX is used for anti-fibrotic effects. Anti-fibrotic efficacy of CBX has previously been shown in the liver and lung fibrosis models (Crespo Yanguas et al., 2018; He et al., 2022). The anti-fibrotic effects of CBX on the MS model have yet to be shown. A study showed that CBX treatment decreases the reactive gliosis and scar formation after a needle-track injury model to the brain (Andersson et al., 2011). Previous studies have shown that in the MOG EAE model, CBX treatment delays the onset of neurological symptoms and decreases the severity (Shijie et al., 2009; Endong et al., 2011; Chen et al., 2020). The authors explain the beneficial effects of CBX by lowering the production of interleukin (IL)-23 and, therefore, the differentiation of T helper (TH)-1 and TH-17, protecting from excitotoxicity by inhibiting glutamate release from microglia and increasing brain-derived neurotrophic factor. In addition, functional loss of Cx43/Cx47 could be associated with spread of chronic MS lesions via the disruption of astrocyte-oligodendrocyte crosstalk, which is important for proper myelination (Basu and Das Sarma, 2018).

The major fibrillar collagens abundantly expressed in liver fibrosis are Collagen 1 and Collagen 3 (Pellicano et al., 2021). We have investigated both ICV and IP CBX groups to evaluate our previous findings further and investigate possible treatment targets by mitigating excessive fibrosis. Both routes of CBX administration improved the clinical scores, and the change in the weight gain was in line with the severity of the neurological symptoms in all groups. We have checked the Collagen 1 and Collagen 3 expression in spinal cord sections and pointed accumulation of Collagen 3 in the PLP group, and prevention of perivascular fibrosis by CBX treatment. One possible explanation for this could be that extensive Collagen 1 accumulation is the hallmark of advanced fibrosis in the liver; whereas, fibrillar Collagen 3 expression starts in the early stages and gradually increases regarding the severity of the disease (Pellicano et al., 2021). We showed that IP CBX did not alter the collagen 3 production but ICV CBX significantly decreased the Collagen 3 accumulation *in-vivo*. This may be due to less access to IP CBX to the brain with low BBB penetrance (Leshchenko et al., 2006). But the protective effect of IP CBX, observed by improvement in clinical scores and decrease in demyelination, points out that multiple mechanisms (systemic inflammation as well as perivascular fibrosis) may be important in disease pathophysiology. Furthermore, incubation with MS sera was able to trigger Collagen and Fibronectin production in the HBVP culture, and CBX was also effective in reducing extensive fibrosis in the *in-vitro* conditions. Notably, MP did not significantly affect the extracellular collagen production on pericytes. This can be explained by the fact that MP exerts its main effects via on immune cells and here only pericytes are studied *in vitro*. Suprisingly, we have seen decreased collagen 3 gene expression, however this change in gene expression did not reflect on extracellular collagen production detected by proline assay and immunofluorescence staining. We can speculate that MP induces various alterations in gene expression in most of the non-immune cells as well.

As previously shown by our group, the MOG and PLP models differ in terms of fibrosis and pericyte behaviors (Şekerdağ-Kılıç et al., 2023). In the current study, the symptoms started at 8 days after immunization in all groups. CBX treatment groups reached the maximum around day 10 and then resolved quickly. On the other hand, in the PLP group, neurological deterioration accelerated after day 14 and continued to increase at the end of the study. The CBX groups showed earlier neurological deterioration, but recovered quickly and remained healthier than the PLP group to the end of the study. Earlier deterioration due to CBX may stem from the BBB dysfunction by the blockage of gap junctions on the BBB-forming cells. Notwithstanding, the quicker recovery after day 12 in the CBX-treated groups might be related to the antifibrotic effects of CBX that have decreased collagen 3 accumulation in this study as well as systemic effects that are studied before (Shijie et al., 2009; Masamune et al., 2013; Chen et al., 2020).

These findings are in line with another article that emphasized the importance of the time window for the therapeutic actions of CBX (Roh et al., 2010). In an intra-hippocampal lipopolysaccharide (LPS) injection-induced neuroinflammation model, CBX has shown to be neuroprotective in the induction phase (1st week) and the healing/regeneration phase (3rd week) but not in the maintenance phase (2nd week) (Anwar et al., 2023). Thus, these results altogether suggest CBX might exert its functions while regulating proinflammatory and anti-inflammatory cytokines, as shown in the MS model in the initial phase, and through glial and perivascular PDGFR positive cells, hindering perivascular fibrosis, oxidative stress, and excitotoxicity in the healing phase (Shijie et al., 2009; Hellmich et al., 2013; Thakur and Nehru, 2015).

Previously, Collagen expression was triggered in hepatic and pancreatic stellate cell cultures incubated with platelet-derived growth factor-BB (PDGF-BB). In that study CBX treatment successfully decreased PDGF-BB-induced cell proliferation, migration, and Collagen 1 expression (Uyama et al., 2003; Masamune et al., 2013). It was confirmed that sera of MS patients also have PDGF-BB (Ulusoy et al., 2021). Hence, in the current study, we showed that the incubation of HBVP with the serum of MS patients significantly increased the expression of Collagen 3, extracellular proline concentration, and fibronectin suggesting increased fibrosis through perivascular PDGFR positive cells. Moreover, all of these were mitigated by CBX, hence underlying the role of anti-fibrotic actions of CBX on pericytes.

The pharmacological anti-fibrotic mechanisms of CBX have yet to be fully discovered (Connors, 2012). For example, the blocking of connexins, especially connexin 43, is thought to be responsible for the actions of CBX in fibrosis (Crespo Yanguas et al., 2018). Connexin 43 loss is associated with a worse prognosis in MS (Masaki, 2015) and also loss of the ability to propagate vasomotor activity by pericytes in diabetic retinopathy (Ivanova et al., 2017). Moreover, pannexin-1 hemichannels are shown to have a putative role in the progression of MS (Ortiz and Puebla, 2020).

Further studies are necessary to reveal whether pannexin or connexin hemichannel blockage results in anti-fibrotic effects of CBX on pericytes and *in vivo* mice model as well as CBX peripheral immunological effects. Hence, the compound that can inhibit fibrosis and inflammation through hemichannels on pericytes can be a novel therapeutic target in MS.

## 5 Conclusion

The contribution of fibrosis at the core of MS lesions and around surrounding vessels to the initiation and progression of MS has remained elusive. Here we demonstrated that an anti-fibrotic agent, carbenoxolone, improves the neurological scores and decreases demyelination in the PLP EAE model and decreases fibrosis in both *in-vivo* and *in-vitro* MS models. Thus, targeting the mechanisms that lead to fibrosis may be a novel therapeutic approach in MS.

## Data availability statement

The original contributions presented in the study are included in the article/supplementary material, further inquiries can be directed to the corresponding authors.

## Ethics statement

The Institutional Review Board (IRB) of Koç University reviewed and approved the use of human serum in *in-vitro* experiments (approval number: 2016.123.IRB2.077). Informed consent was obtained from all subjects, and the study has been performed in accordance with the declaration of Helsinki. The animal study was approved by the Institutional Animal Care and Use Committee (IACUC) at Koç University (2016-02, 2022-11 approval numbers). The study was conducted in accordance with the local legislation and institutional requirements.

## Author contributions

EU: Data curation, Formal analysis, Investigation, Methodology, Validation, Writing – original draft, Writing – review & editing. EO: Conceptualization, Data curation, Investigation, Methodology, Supervision, Writing – original draft. NS: Investigation, Methodology, Validation, Writing – original draft, Data curation. ES-K: Conceptualization, Data curation, Investigation, Methodology, Writing – original draft. FA: Conceptualization, Data curation, Investigation, Methodology, Validation, Visualization, Writing – original draft. SS: Conceptualization, Data curation, Investigation, Methodology, Supervision, Writing – original draft, Writing – review & editing. JK: Data curation, Investigation, Methodology, Writing – original draft. CO: Data curation, Investigation, Methodology, Writing – original draft. AB: Data curation, Methodology, Writing – original draft. YG-O: Conceptualization, Formal analysis, Funding acquisition, Investigation, Methodology, Resources, Supervision, Validation, Writing – original draft, Writing – review & editing.
